# Effect of using immobilization device in fluoroscopic study in pediatric patient: Focused on radiation dose reduction in voiding cystourethrogram

**DOI:** 10.1371/journal.pone.0224063

**Published:** 2019-10-18

**Authors:** Hyun-Hae Cho, So Mi Lee, Sun Kyoung You

**Affiliations:** 1 Department of Radiology, Ewha Womans University Mokdong Hospital, Seoul, Republic of Korea; 2 Department of Radiology, Kyungpook National University Hospital, Daegu, Republic of Korea; 3 Department of Radiology, Chungnam National University Hospital, Daejeon, Republic of Korea; All India Institute of Medical Sciences, Bhopal, INDIA

## Abstract

**Introduction:**

To prove objective effect of using mechanical device for immobilization of pediatric patient during voiding cystourethrogram (VCUG) compare immobilization by hand-holding.

**Methods:**

This study included 77 patients, who underwent VCUG in our center from April to October 2017, who had a clinically suspicious urinary tract infection. Patients were classified into one of two groups based on whether examination was done before (Group A) or after (Group B) adaptation of immobilization device. Patient-related data, image quality related score and dose-related data were collected and compared between two groups.

**Results:**

Group A included 36 patients and group B included 41. Patient related data including mean age, sex, body weight and height didn’t show significant difference between two groups (*p >* 0.05 for all). Among the image quality scoring, overall image quality, motion artifact, showed significant difference between two groups with improved inadequate timing and centering after adaptation of immobilization device. Dose related data showed significantly decreased shot number, mean fluoroscopic time with decreased mean dose area product (DAP) value and effective dose after adaptation of immobilization device (*p <* 0.05 for all).

**Conclusion:**

Adaptation of immobilization device can improve overall image quality with decreased motion artifact and improved centering and timing with even shot number, mean fluoroscopic time with decreased mean DAP value and effective dose.

## Introduction

Fluoroscopy examination is one of widely used diagnostic method in pediatric patients, because they can provide information about peristalsis or reflux in real time [1]. Although uncontrolled radiation exposure can occur during fluoroscopy due to relatively high patient and operator dependency on the fluoroscopy procedure. And pediatric patients, who are more sensitive to radiation exposure than adults [2–4], there should be more attention devoted to fluoroscopy procedures performed in pediatric patients.

Previous studies have offered recommendations to reduce exposure through modification of the technical aspects of fluoroscopic examination, such as reduction of pulse rate, grid removal and minimization of full-exposure images, improvement of collimation and reduction in fluoroscopy duration [1, 5, 6]. Immobilization is especially important in young, uncooperative patients because all imaging studies are based on still images. Immobilization is achieved using many different methods including hand-holding by a parent and/or technologist, or using other mechanical restraint devices [7].

Although immobilization is believed to be an important part of imaging studies in pediatric patients [7], to our knowledge, no previous studies have objectively compared immobilization tools. Accordingly, this study aimed to compare immobilization by hand-holding and a mechanical device during voiding cystourethrogram (VCUG), which represents one of the most common fluoroscopic examinations performed in pediatric patients [1, 5, 6, 8].

## Materials and methods

Institutional review board of Ewha Womans University Mokdong Hospital approved the study and written informed consent was obtained.

### Patients

From April to October 2017, a total of 114 pediatric patients underwent VCUG in the authors’ department. Among them, 5 patients were excluded because they were > 3 years of age and were able to obey verbal commands for positioning during fluoroscopic evaluation. Ten patients were excluded because their studies were performed for follow-up purposes, in which the follow-up protocol differed from the initial protocol. The authors do not acquire pre-contrast filling anteroposterior (AP) and AP images after voiding. An additional 7 patients were excluded due to a lack of permission/consent. One patient was excluded because there was underlying complete duplication of urinary collection system. It was, therefore, necessary to acquire additional images for evaluating ureterocele and reflux that did not use the routine VCUG protocol.

Finally, a total of 77 patients (37 female, 40 male; mean age, 6.1 months [range, 3 to 18 months]) were included in this study. Thirty-six patients underwent study before adaptation of the immobilization device, while 41 underwent study after implementation of a immobilization device.

### Routine protocol

All procedures were performed by a pediatric radiologist (H.H.C [> 7 years’ experience in pediatric radiology]) who was assisted by a junior resident and a radiation technologist.

All the examinations were performed using two digital fluoroscopy systems (Sonialvision Safire II and 17, Shimadzu, Kyoto, Japan) with baseline settings of 150 kVp and 80 mAs, with a field of view (FOV) from 9–7 inches, without application of automatic exposure control (AEC). The frame rate was set to 30/3.5 sec and focal spot-film distance ranged approximately 1100–1500. DICOM type imaging intensifier format was adapted. Antiscatter grids were not used for this age group of patients.

VCUG was performed by experienced personnel based on the guidelines for VCUG [9]. Aseptic catheterization of the urinary bladder (UB) was performed using a 3 Fr or 4 Fr Nelaton catheter based on patient age and size. Positioning of the catheter within the UB was performed according to whether there was natural drainage of urine. Kinking or knotting of the catheter was avoided by not inserting an unnecessarily long catheter within the UB. After positioning of the Nelaton catheter within the UB, contrast material (Iopamidol, Pamiray 300, Dongkook Pharmaceutical, Seoul, South Korea) was infused to fill the UB by gravity drip. The total amount of contrast material used for full filling the UB was approximately 30 to 50 mL, and was determined according to age and body weight of the patient. If a sudden decrease in flow of the contrast material during the infusion was noted, infusion was stopped, even if the UB was insufficiently filled. Subsequently, intermittent fluoroscopic verification for the existence of UB lesions, such as neurogenic bladder, ureterocele, vesicoureteral reflux (VUR) with or without hydroureteronephrosis before voiding, was performed. When the patient began to void, verification of VUR, with or without hydroureteronephrosis, retrograde renal staining, and urethral abnormality was performed. After voiding was complete, residual urine or vesicovaginal reflux was verified.

**Fig 1:**
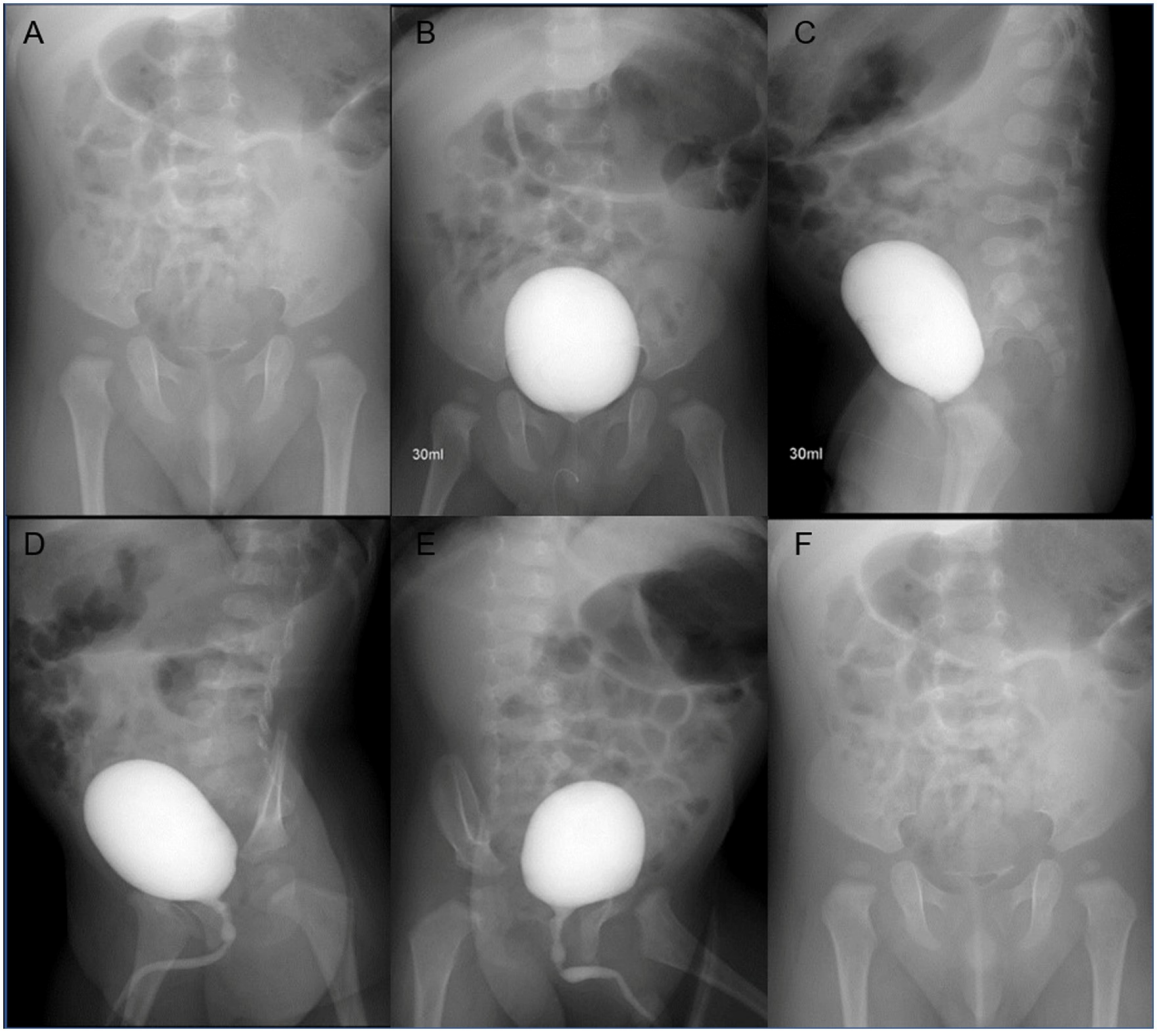
Routine protocol of VCUG in our center. (A) A pre-contrast filling anteroposterior (AP) image. (B) An AP image with a contrast-filled UB. (C) A lateral image of the contrast-filled UB. (D) and (E) Right and left oblique image during voiding. (F) An AP image after voiding. VCUG, voiding cystourethrogram.

The routine VCUG protocol used at the authors’ center includes 6 essential scout images for more objective evaluation: a pre-contrast filling AP image; an AP image with contrast-filled UB; a lateral image of the contrast-filled UB; a right and left oblique image during voiding; and an AP image after voiding (Fig 1). For complete evaluation of both the renal pelvis and urethra, the FOV of the scout image included the kidney to the pubic bone.

During all these procedures, immobilization of pediatric patients was required. None of these patients, however, underwent sedation. Before implementation of a commercially available immobilization device, a technologist and the parents of the child directly fixed the child with both hands held in a raised position above the head. After adaptation of the immobilization device, there was no additional need for manual fixation.

### Immobilization device

There are many available customized immobilization devices for radiological examination of difficult-to-control pediatric patients. Among them, the Octostop and Octoroll unit (OCTOSTOP Inc. Montreal, Canada) was used in this study. The Octostop is composed of a plate and a fixing cloth, while the Octoroll consists of a wheel and a regulator that can rotate 360 degrees. Using this unit, it is possible to immobilize and position the patient at the same time. Positioning is an especially important part of fluoroscopic examinations because the posture of the child must change continuously during the fluoroscopic examination to acquire proper images to make an accurate diagnosis.

### Data collection

Patient-related data, including age, sex, body weight and height on examination day, were collected. The clinical history of patient included diagnosis, laboratory findings, history, and ultrasonography findings; the results of VCUG was also collected.

Image quality was reviewed by consensus of 3 pediatric radiologists (H.H.C, S.M.L, and S.K.Y [7 years’ experience each]), who were blinded to clinical history and other imaging findings, as well as the final diagnosis of the patients. All image data sets were displayed on a picture archiving and communication systems (PACS) workstation.

**Fig 2:**
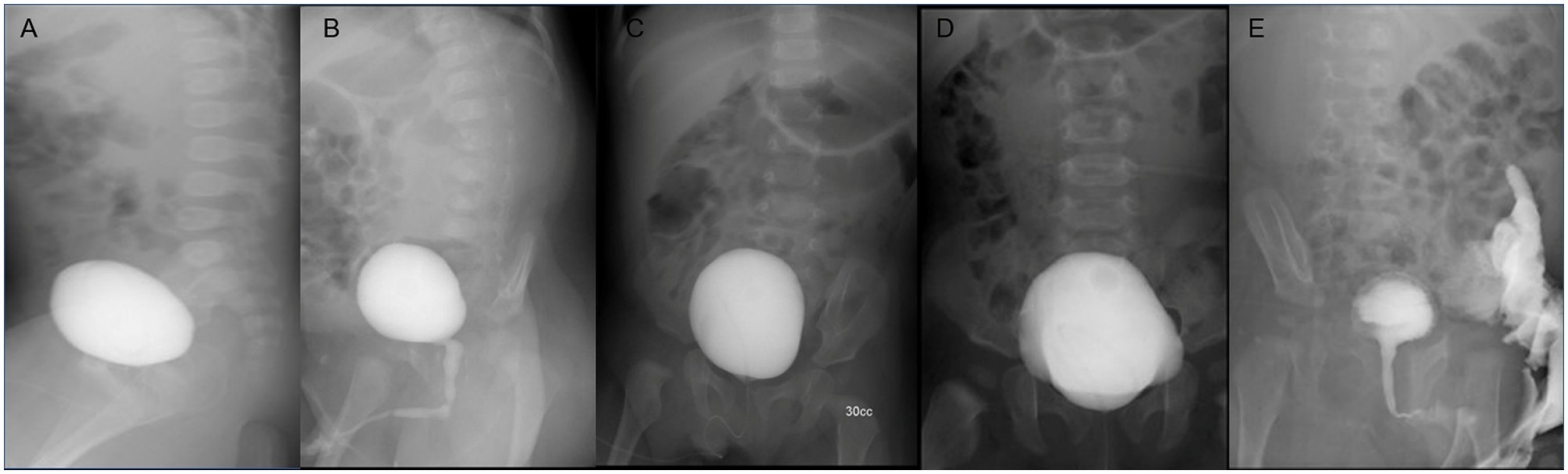
Examples for inadequate centering and timing. Abnormal tilted positioning with deviation of spine axis and tilted pelvis on lateral (A) and oblique image (B) with blurred bony structures due to motion artifact. (C) and (D) show abnormal centering of contrast filled urinary bladder. (E) External artifact noted by contrast excretion.

Overall image quality was graded on a 5-point scale: 1, unacceptable; 2, poor; 3, average or acceptable; 4, good; and 5, excellent or ideal. Motion artifacts, external artifacts, inadequate shoot timing and inadequate centering were graded as 0 for absent and 1 for present (Fig 2).

It was difficult to determine discomfort of the patients during examination due to their young age. Irritability was scored during the examination on a 3-point scale based on the operator’s discretion for estimation of discomfort: 1, stable during the examination; 2, moderate irritability noted but tolerable and did not interfere the examination course; and 3, severe irritability noted.

These data were compared between the two groups (i.e., hand-holding [manual fixation] without an immobilization device [group A]; or immobilization device [group B]).

### Dose related data

Dose-related data, including the number of images acquired, total examination time and fluoroscopic time, mean kVp and mAs, and total dose area product (DAP) value were collected. Dose-related data were collected based on the installed DAP meter (Vacu DAP, Dresden, Germany).

Although DAP itself can be appropriate to estimate radiation dose in a fluoroscopy procedure [10], the effective dose was calculated in two different ways. Initially, a conversion factor was calculated using monte carlo simulation via PCXMC version 1.5 (STUK, Helsinki, Finland). One million photon histories were followed with approximately 4% associated standard error. The derived conversion factor was approximately 0.18 mSv/Gy.cm^2^. Alternatively, a published conversion factor 0.21 mSv/Gy.cm^2^ was used for calculation [11].

### Statistical analysis

To compare the two groups, the independent samples t-test and Fisher’s exact test were used in SPSS version 20.0 (IBM Corporation, Armonk, NY, USA); *p* < 0.05 was considered to be statistically significant.

## Results

### Patient-related data

A total of 77 patients (37 female, 40 male; mean age, 6.1 months [range, 3 to 22 months]) were included in this study. Thirty-six patients (group A: 17 female, 19 male; mean age, 5.8 months [range, 3 to 21 months) underwent the study by hand-holding before implementation of the immobilization device, and 41 (group B: 21 female, 20 male; mean age, 6.4 months [range, 3 to 22 months]) underwent study after implementation of the immobilization device. There were no significant differences between the two groups in age, sex distribution, body weight, or height on examination day (Table 1).

**Table 1:** Comparison of patient related factors between the two groups.

	Group A (n = 36)	Group B (n = 41)	*P -value*
**Mean age (mons)**	5.8	6.4	0.570
**Sex (male)**	19 (53.2%)	21 (54.0%)	0.896
**Body weight (kg)**	7.8	7.9	1.000
**Height (cm)**	69.0	68.4	1.000

All patients complained of fever, which was combined with diarrhea in 5 individuals. Nine patients complained of cough. None of these patients, however, had an abnormal clinical history.

Among the 77 patients, 70 were clinically diagnosed with a urinary tract infection (UTI) and 7 had findings suggestive of hydroureteronephrosis. Leukocytosis was noted in 60 patients, with elevated levels of C-reactive protein in 7. All patients exhibited positive white blood cell count on urine analysis with positive urine culture. Proteinuria was noted in 3 patients.

All VCUG was performed after screening ultrasonography, and the mean duration between US and VCUG was approximately 3.2 days (range, 2 to 6 days) after acute phase of the disease. US results demonstrated UTI involvement in all patients, and acute pyelonephritis was suggested in 47 patients, with combined hydroureteronephrosis noted in 16 patients.

Results of VCUG revealed ipsilateral VUR in 20 patients: grade I (n = 1), grade II (n = 6), grade III (n = 8), grade IV (n = 3); and grade V (n = 2). Bilateral VUR was noted in 8 patients: grade II on right and grade I on left (n = 2); grade III on right and grade II on left (n = 4); and both grade III (n = 2).

### Image quality-related data

Overall image quality scored significantly higher in group B than in group A (*p* < 0.001). Motion artifacts were significantly higher in group A than in group B (*p* = 0.001). External artifacts did not demonstrate significant difference between two groups (*p* > 0.005). Inadequate shoot time was noted significantly more often in group A than group B (*p* = 0.031). Inadequate centering was also noted significantly more often in group A than group B (*p* = 0.011).

Scoring for patient irritability did not demonstrate a significant difference between the two groups (p > 0.005). A comparison of the image quality-related data is presented in Table 2.

**Table 2:** Comparison of image quality related factors between the two groups.

	Group A (n = 36)	Group B (n = 41)	*P -value*
**Overall image quality**	3.9	4.8	0.000*
**Motion artifact**	14	3	0.001*
**External artifact**	8	6	0.392
**Inadequate timing**	6	1	0.031*
**Inadequate centering**	15	4	0.011*
**Discomfort**	1.8	1.9	0.889

Note:

*Mean value is significant (P < 0.05) different between two groups.

### Dose-related data

The number of scout images acquired during VCUG was approximately 9.8 in group A and 8.1 in group B, which was a statistically significant difference (*p* = 0.018). Mean kVp and mAs of the scout images did not demonstrate significant differences between two groups (*p* > 0.005).

The mean duration of fluoroscopy was significantly longer in group A than in group B (*p* = 0.013). Mean kVp and mAs of the fluoroscopic study did not demonstrate a significant difference between two groups (*p* > 0.005). The mean DAP was measured to be significantly higher in group A than in group B (*p* = 0.001). The mean effective dose calculated by using conversion factors, derived from calculation and reported conversion factor was significantly higher in group A than in group B (p = 0.001). Dose-related data comparisons are shown in Table 3.

**Table 3:** Comparison of dose related factors between the two groups.

	Group A (n = 36)	Group B (n = 41)	*P -value*
**Shot number**	9.8	8.1	0.018*
**Mean KVP of shot**	76.1	76.2	0.845
**Mean mAs of shot**	6.8	6.4	0.221
**Mean time of fluoroscopy**	191.9	152.9	0.013*
**Mean KVP of fluoroscopy**	70.4	70.4	0.995
**Mean mAs of fluoroscopy**	2.9	3.6	0.240
**Mean Dose (DAP)**	619.5	312.8	0.001*
**Mean Effective Dose** ^1^	1.1	0.6	0.001*
**Mean Effective Dose** ^2^	1.3	0.7	0.001*

Note:

*Mean value is significant (P < 0.05) different between two groups.

^1^ Result is calculated by using conversion factor derived from monte carlo simulation.

^2^ Result is calculated by using conversion factor derived from published data.

## Discussion

The results of this study revealed a decreased shot number during VCUG, which is the most common fluoroscopy procedure performed in pediatric patients. Decreased mean fluoroscopic duration was also noted and resulted in a reduction of radiation dose exposure during the examination. Decreased incidence of motion artifacts and inadequate shot timing and centering can improve overall image quality in pediatric VCUG studies by using immobilization devices.

Radiation exposure is one of the most important issues in imaging studies involving pediatric patients [1, 5, 12]. Although fluoroscopic studies have played a major role in diagnosing many pediatric diseases [3–5], there has been relatively little effort devoted to reducing radiation exposure during the examination [6]. It is difficult to estimate and calculate the radiation dose exposure during the procedure due to variable procedural protocols among patients, operators, and centers [3, 4, 12–14]. Therefore, there is significant variation in fluoroscopic duration or number of scout image shots among different centers. Moreover, there is even a noted deviation among operators within the same centers [3, 4, 12–14].

Generally, many tips, which were provided by pause and pulse campaign, is used for reducing radiation for pediatric fluoroscopic studies including recommendation of pulsed fluoroscopy, grid removal, and last image hold [3, 4]. However, these methods can be complicated to adapt and require additional education and/or training. Therefore, we focused on a simpler way to reduce radiation dose by using an immobilization device. Immobilization is easy to adapt and is a generally used method for uncooperative pediatric patients, and essential for exact imaging evaluation.

Immobilization becomes an issue in computed tomography or magnetic resonance imaging due to the relatively long examination times of these imaging methods compared with simple radiography, and concerns regarding sedation of pediatric patients for imaging studies [7, 15]. We do not use sedation for any fluoroscopy procedure in pediatric patients; nevertheless, the remaining issue of relatively long examination times, which is dependent on the patient and operator, remains. To this point, fluoroscopic studies can cause unnecessary radiation exposure to pediatric patients because the irritability of pediatric patients is not predictable and is generally uncontrollable, especially in the absence of sufficient experience and background knowledge, which can prolong the duration of the procedure under circumstances involving an irritable patient. As fluoroscopy duration increases, the radiation dose increases accordingly [1, 16].

Motion artifacts during an imaging study can make it difficult to acquire proper image quality for accurate evaluation. Furthermore, they can cause repetitive shooting and study for more information, which cause excessive radiation exposure to pediatric patients. A previous study reported that motion, positioning, or inappropriate exposure are the most common causes of excessive radiation exposure [17]. From this perspective, reduction of motion artifacts by using immobilization can be one solution toward radiation dose reduction [18]. Results of this study support the use of an immobilization device reflected by the significant reduction in motion artifacts.

During VCUG, a conventional hand-holding method for immobilization could not prevent the movement of the abdomen and pelvic area because we held the patient’s upper and lower extremity for limiting motion and positioning. Motion of those areas can be the main cause of motion artifacts because those areas are main target area for VCUG. By using an immobilization device, however, we were able to prevent motion in these areas using radiolucent Velcro belts, which can restrain the abdomen and pelvis without affecting stored images.

Aside from the essential positive effect of implementation of immobilization devices, other aspects should also be considered. The safety of pediatric patients restrained by immobilization devices is an essential precondition for using these devices. Distress of pediatric patients who undergo fluoroscopic studies with immobilization devices should also be considered [2, 18]. To reduce these limitations, commercially available immobilization devices that have undergone a sufficient validation process should be used. We had concerns about increasing irritability in our pediatric patients due to uncomfortable positioning and the sensation of the immobilization device; however, our results demonstrated no significant increased irritability, which supports the use of immobilization devices. We also recommend making the fluoroscopy procedure more familiar to pediatric patients by attendance of their parents in the examination room, and using toys, music, and movies to comfort them in unfamiliar situations [2, 7].

Virtually all studies regarding fluoroscopic procedures share the common problem of patient and operator dependency in all parts of the fluoroscopic procedure. Therefore, some variability may exist among procedures, and this variability can cause bias. To mitigate this, our hospital uses training and testing methods according to a standardized protocol. Therefore, we propose that our results are sufficiently robust to demonstrate a trend. Initially, we believed that an immobilization device could lead to some reduction of scattered radiation because it could completely replace the need for a technologist, operator, or parents to stand beside patients undergoing fluoroscopic procedures. However, in addition to providing a more comfortable and familiar environment for pediatric patients, verbal sedation and eye contact by a technologist or parents, who stand beside the patient, is still needed.

The results of the present study demonstrated a decreased shot number during VCUG, which is the most common fluoroscopy procedure performed in pediatric patients. Decreased mean fluoroscopic duration was also noted and resulted in a reduction of radiation exposure during the examination. Decreased incidence of motion artifacts and inadequate centering can improve overall image quality in pediatric VCUG studies by using an immobilization device. Therefore, we recommend using an immobilization device for pediatric patients who undergo fluoroscopic procedures. Additional studies are needed for other types of fluoroscopic procedures, such as colon or upper gastrointestinal studies, to further validate the use of immobilization devices in common pediatric fluoroscopic procedures.

## Conclusion

Using the immobilization device for VCUG can resulted in reduction of radiation exposure during the examination by reducing the motion artifact and inadequate centering which could cause poor image quality or repetitive examination in pediatric patients. In this point, using the immobilization device can be one of effort for reduction of radiation exposure during fluoroscopic evaluation in pediatric patients.

## Supporting information

S1 Table Table including fluoroscopic, image quality and clinical data of each group. (XLSX)
